# A frailty index predicts post-liver transplant morbidity and mortality in HIV-positive patients

**DOI:** 10.1186/s12981-017-0163-x

**Published:** 2017-08-05

**Authors:** Giovanni Guaraldi, Giovanni Dolci, Stefano Zona, Giuseppe Tarantino, Valentina Serra, Roberto Ballarin, Erica Franceschini, Mauro Codeluppi, Thomas D. Brothers, Cristina Mussini, Fabrizio Di Benedetto

**Affiliations:** 1 Department of Medical and Surgical Sciences for Adults and Children, Clinic of Infectious Diseases, University of Modena and Reggio Emilia, Largo del Pozzo, 71, 41124 Modena, Italy; 2 Department of Medical and Surgical Sciences for Adults and Children, Liver and Multivisceral Transplant Center, University of Modena and Reggio Emilia, Modena, Italy; 3 Faculty of Medicine, Dalhousie University, Halifax, B3H 2E1 Canada

**Keywords:** Frailty, HIV, Transplant

## Abstract

**Background:**

We hypothesized that frailty acts as a measure of health outcomes in the context of LT. The aim of this study was to explore frailty index across LT, as a measure of morbidity and mortality. This was a retrospective observational study including all consecutive 47 HIV+patients who received LT in Modena, Italy from 2003 to June 2015.

**Methods:**

frailty index (FI) was constructed from 30 health variables. It was used both as a continuous score and as a categorical variable, defining ‘most frail’ a FI > 0.45. FI change across transplant (deltaFI, Δ*FI*) was calculated as the difference between year 1 FI (FI–Y1) and pre-transplant FI (FI–t0). The outcomes measures were mortality and “otpimal LT” (defined as being alive without multi-morbidity).

**Results:**

Median value of FI–t0 was 0.48 (IQR 0.42–0.52), FI–Y1 was 0.31 (IQR 0.26–0.41). At year five mortality rate was 45%, “optimal transplant” rate at year 1 was 38%. All the patients who died in the post-LT were most frail in the pre-LT. Δ*FI* was a predictor of mortality after correction for age and MELD (HR = 1.10, p = 0.006) and was inversely associated with optimal transplant after correction for age (HR = 1.04, p = 0.01).

**Conclusions:**

We validated FI as a valuable health measure in HIV transplant. In particular, we found a relevant correlation between FI strata at baseline and mortality and a statistically significant correlation between, Δ*FI* and survival rate.

**Electronic supplementary material:**

The online version of this article (doi:10.1186/s12981-017-0163-x) contains supplementary material, which is available to authorized users.

## Background

Liver transplantation (LT) is theoretically the best treatment for patients with end stage liver disease (ESLD) but its effectiveness is limited by short-term mortality and morbidity, and by persistent shortage of donors’ organs. Specific selection policies have been developed to identify good candidates for this surgical option [1, 2]. In this context, recipients with chronic health conditions, including HIV [3–6], can be admitted to this surgical procedure after appropriate selection.

Under the current medical urgency-based selection system, patients with worst outcomes while on the waiting list, are given highest priority for LT [7] using model for end-stage liver disease (MELD) score [8], acting as a predictor of mortality in waiting list period. Unluckily this algorithms fails to depict the extremely relevant heterogeneity of morbidity and quality of life of patients in the post-LT period [9, 10]. In addition, in HIV settings, and in particular in HIV/HCV co-infected patients, the prognostic value of the MELD score is uncertain. Some studies [11, 12] found a significant correlation between MELD and post-LT mortality, whereas in others this model had limited prognostic value [13, 14].

Frailty, conceptualized as frailty phenotype [15] or as an accumulation of deficit measure in a Frailty Index (FI) score [16], has the potential to predict relevant post-LT outcomes and assess the overall health status of the candidates.

Frailty phenotype in the HIV negative solid organ transplant population has been proven to be associated with increased mortality, early hospital readmission, delayed graft function after kidney transplant [17–20] and poor health outcomes in liver disease patients [21–24], including LT waitlist mortality [24].

Derck et al. [23], recently showed that frailty phenotype outperformed MELD in predicting quality of life in the pre-transplant period.

Moreover, Wilson et al. [25] demonstrated the predictive power of pre-transplant FI on post-transplant mortality in the setting of lung transplant.

Finally, Lai et al. [26] showed an association between measures of physical function related to frailty and post-LT mortality.

Frailty Index (FI) describes a multidimensional risk state which summarizes health deficits across a range of symptoms, signs, diseases, disabilities and laboratory abnormalities [16, 27]. This approach is robust across different settings, in different populations, using different numbers and types of health variables, consistently related to age and to adverse outcomes. Notably, this predictive capacity transferred into a scale measure (index) can be used in clinical practice to monitor health transition over time and in relation to medical interventions [28].

Our group have previously validated a FI in HIV-positive patients to measure biological aging, health status and prediction of mortality and multi-morbidity (MM) incidence [29, 30]. We decided to expand this experience in the context of LT in HIV patients. In this peculiar setting patient selection is more stringent due to the higher risk of morbidity and mortality of this vulnerable population [31].

We hypothesized that frailty, conceptualised as a deficit accumulation, acts as a measure of health outcomes that covers the overall LT process.

The aim of this study was to explore frailty index (FI) across LT, as a measure of morbidity and mortality.

## Methods

Retrospective observational study including all consecutive HIV infected patients who received cadaveric donor liver transplant at the Liver and Multivisceral Transplant Center of University of Modena and Reggio Emilia from June 2003 to June 2015.

Baseline data included demographics (ethnic origin, gender and age), HIV characteristics (transmission risk factors, duration of HIV infection, HIV CDC classification, nadir CD4 cell count, class type and exposure to antiretroviral therapy, plasma HIV genotypic resistance assay antecedent or concomitant to switching and current HAART) and ESLD history (aetiology, known duration of cirrhosis, HCV genotype, baseline serum HCV–RNA VL, previous cirrhotic decompensation episodes and presence of histologically documented hepatocellular carcinoma).

Donor risk index (DRI), comprising the following variables: donor age, donation after cardiac death, split/partial grafts, African-American race, less height, cerebrovascular accident and ‘other’ causes of brain death, location and cold time, was retrospectively calculated as a characteristics associated with liver graft failure but was not a graft allocation criteria [32].

### Outcomes

Outcome measures considered in this cohort were:

1. Mortality: vital status was regularly updated at Liver and Multivisceral Transplant Center of University of Modena and Reggio Emilia via telephone contact and hospital records.
2. Optimal transplant: per protocol was defined as the post-LT survival of the patient in the absence of multi-morbidity. Multi-morbidity (MM) was described as the presence of three or more of the following six comorbidities: cardiovascular disease (clinical diagnosis with history of myocardial infarction, stroke, revascularization, or peripheral artery disease); hypertension (blood pressure measured twice ≥140 mmHg systolic or ≥90 mmHg diastolic or taking antihypertensive medicine); type two diabetes mellitus (measured fasting glucose ≥126 or oral glucose tolerance test >200 or on treatment); chronic kidney disease (estimated glomerular filtration rate <60 mL/min, via Modification of Diet in Renal Disease study equation [33]); osteopenia (dual-energy X-ray absorptiometry t or z score <−1.5) or osteoporosis (dual-energy X-ray absorptiometry t or z score <−2.5 or fragility fracture); or dyslipidemia (total cholesterol >200 mg/dL or low-density lipoprotein >100 mg/dL or high-density lipoprotein >100 mg/dL or triglycerides >150 mg/dL). COPD was not included as it is an exclusion criteria for liver transplant [34].

Non-optimal transplant was defined as the absence of optimal transplant, due to mortality or MM.

### Predictors

Frailty index: was built, as previously described [29] to calculate the proportion of health deficits individuals have accumulated out of a group of 30 relatively nonspecific health variables (Table 1). Each variable included was recorded with values of one when a deficit was present, and zero when absent [35]. Missing values were removed from both numerator and denominator of the FI (FI = $\frac{\mathop \sum \nolimits Deficit}{30 - \mathop \sum \nolimits Missingvalues}$).

**Table 1:** Variables included in the Frailty Index

Nr.	Variable	Deficit description
1	Hypertension	Measured blood pressure or on treatment
2	Diabetes mellitus type II	Fasting glucose >125 mg/dL or on treatment
3	Chronic kidney disease	Two estimated glomerular filtration rate measurements <60 mL/min/1.73 m^2^
4	Osteopenia/Osteoporosis	Osteopenia: dual-energy X-ray absorptiometry (DEXA) Tor Z score <−1.5 or Osteoporosis: DEXA Tor Z score <−2.5 or fragility fracture
5	Any cancer	Clinical diagnosis
6	High or low BMI	<18 or >25 kg/m^2^
7	High total cholesterol	>200 mg/dL
8	High triglycerides	>150 mg/dL
9	Leucopenia	<4000 cells/µL
10	Anemia	If female: <10 g/dL If male: <12 g/dL
11	Low platelets	<150 billion/L
12	Hyponatremia	<125 mEq/L
13	Abnormal potassium	<3.5 or >5.3 mEq/L
14	Elevated aspartate transaminase	>31 U/L
15	Elevated alanine transaminase	>31 U/L
16	Abnormal alkaline phosphatase	<38 or >126 U/L
17	Elevated γ-glutamyl transphosphatase	>55 U/L
18	Elevated total bilirubin	>1.10 mg/dL
19	Elevated creatinine	If female: >1.0 mg/dL If male: >1.2 mg/dL
20	Elevated INR	>1.2
21	Hepatits C virus viral load	HCV-RNA >12 copies/mL
22	Hepatitis B coinfection	Pre-LT: positive HBeAg Post-LT: HBV-DNA >copies/mL
23	Epstein-Barr virus coinfection	Pre-LT: positive EBV IgG Post-LT: HBV-DNA >copies/mL
24	Cytomegalovirus coinfection	Pre-LT: CMV IgG Post-LT: HBV-DNA >copies/mL
25	HIV viral load	>40 copies/µL
26	Current CD4^+^ cell count	<500 cells/µL
27	CD4^+^/CD8^+^cell ratio	<1
28	Nadir CD4^+^ cell count	<200 cells/µL
29	History of AIDS	History of CDC category C HIV disease [38]
30	Duration of HIV infection at LT	>20.6 years

Study visits were applicable for FI evaluation if they had at least 80% of available health variables at that visit.

According to literature [36], we classified as “most frail” in a particular moment patients with FI > 0.45, as “frail” persons with FI between 0.21 and 0.45 and as “fit” or “not frail” people with FI < 0.21.

It must be noticed that in the risk prediction of “optimal/non-optimal transplant” a modified version of FI not listing the 6 comorbidities used to define MM, was used to avoid overlap between predictive variables and study outcome.

Frailty was assessed pre-LT (baseline) and at year 1, 3 and 5. Patients who died or did not reach year 1 post-LT censored FI data at last available assessment.

Pre-transplant FI (FI–t0) was calculated at the last available data before transplant (baseline visit), within 30 days before LT.

Post-LT FI was assessed at a median period of 262 days (IQR = 87–324) at year 1 visit (FI–Y1), at a median period of 1090 days (IQR = 1028–1142) at year 3 visit (FI–Y3) at a median period of 1843 days (IQR = 1800–1877) at year 5 visit (FI–Y5).

FI change across transplant ( Δ*FI*), was calculated as the score difference between FI–Y1 and FI–t0.

$$\Delta FI = FIY1 - FIt0$$

We assessed FI validity using a three part approach that comprehends content, construct, and criterion validity as previously described [29, 37].

Other predictors at transplant time were: donor risk index, gender, age, BMI, MELD HCV antibody and RNA detectability, CD4 nadir and current, history of AIDS conditions and HIV viral load.

### Statistical analysis

Descriptive statistics and FI scores at the baseline were calculated and distributions visualized. Cross-sectional relationships between FI scores and years of age were evaluated by linear regression. For prediction models, frailty index score was categorized using 0.1 increment. Each covariate was evaluated in univariate prediction models due to the small sample size and the low rate of events.

- Mortality: Kaplan–Meier approach was used to evaluate mortality. Univariate Cox regression analysis was used to assess factors associated with mortality. We also calculated mortality in the 3 categorical frailty strata.
- Unsuccessful LT: Multivariate analysis was used to assess the association between FIt0 and non-optimal transplant, FIY1 and non-optimal transplant, correcting for age. A Univariate Poisson analysis was used to assess the association between the 3 frailty strata at the baseline and non-optimal transplant.
- Health transition after LT: FI was used to evaluate health transition after LT. LOWESS smoother graphs were drawn to describe FI over time. A linear regression model was used to determine the association between FI–t0 and, Δ*FI* at the year 1, year 3 and year 5 visit. The same method was used to determine the association between FI–t0 and FI–Y1.

## Results

### Population characteristics at baseline

Forty-seven HIV-positive patients underwent LT at Liver and Multivisceral Transplant Center of University of Modena and Reggio Emilia since from 2003 to June 2015. Among them, 5 underwent simultaneous transplant of liver and kidney.

Most LT patients were males (80.8%) with a median age of 51.2 years; IQR = 45.91–54.35).

Median pre-transplant CD4^+^ count was 260 cells/µL (IQR 153–360 cells/µL), with only 9 patients (17%) having a CD4 count above 500 cells/µL. Most of the patients (44; 93.6%) presented undetectable HIV-RNA at transplant time.

Median baseline frailty index was 0.48 (IQR 0.42–0.52).

### Liver transplant indications

The most common indication for liver transplant was HCV-induced ESLD (82.9%), followed by hepatocellular carcinoma (HCC; 44.6%). Twenty-eight patients presented multiple indications, in particular, two of them had four indications (association of HCV, HBV, HDV and HCC in both cases), six had three indications and 20 had two indications. Among this latter group, the most prevalent association (60%) was HCV and HCC. Median pre-transplant MELD was 22 (IQR 16.5–29).

### Mortality

Mortality at year five post-LT was 45%, while at year 1 and year 3 was respectively 21 and 38%.

A multivariate analysis including all baseline FI variables was performed, but none of them reached statistical significance in predicting post-transplant mortality.

**Fig. 1:**
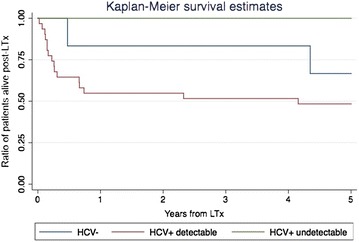
Kaplan–Meier survival estimate in relation to HCV RNA at the time of transplantation

Figure 1 describes how mortality was related to HCV antibody status and HCV RNA detectability at transplantation. Of interest, seven HCV + patients were treated in the pre-LT with HCV directly active agents (DAA) and none of them died in the 1-year follow up.

### Optimal transplant/Non-optimal transplant

Prevalence of MM was fairly stable in the post-LT period, respectively prevalent in 39, 46 and 38% at year 1, 3 and 5.

As reported in Additional file 1: Figure S1 the proportion of survivors with no MM, who met the definition of “Optimal transplant”, were respectively 38% (17 patients) at year 1, 29% (10) at year 3 and 26% (8) at year 5.

### Frailty index

#### Construct validity: frailty index description

Pre-transplant frailty index ranged from 0.32 to 0.62, with a median of 0.48 (IQR 0.42–0.52). Consistently with literature [29, 35, 38, 39], its upper limit was close to 0.7. It was significantly associated with age (r = 0.30, p = 0.048) and followed a normal distribution. At baseline 29 patients (61.7%) were classified as “most frail”, whilst 18 (38.3%) were “frail” and no one was “not frail”.

No significant correlation was observed between baseline FI and pre-transplant MELD (r = 0.14, p = 0.932). Pre-LT frailty index was conversely correlated with donor risk index [32] (DRI) (r = −0.50; p = 0.008).

#### Criterion validity: DELTA FI ability to predict major outcomes

Baseline FI, analysed into 0.1 increment was not significantly associated with survival (HR = 1.47; p = 0.90). However, all the 10 deceased patients (100%) at year 1 were “most frail” at baseline (HR and p value are not statistically applicable in this situation).

FI at year 1 was associated with mortality after correction for age (HR = 1.12, p = 0.04) or for FIt0 (HR = 1.13, p = 0.03).

Univariate Poisson regression analysis of the association between non-optimal transplant and frailty strata at the baseline showed an IRR = 1.45 (CI  0.7078–2.98; p = 0.3).

**Fig. 2:**
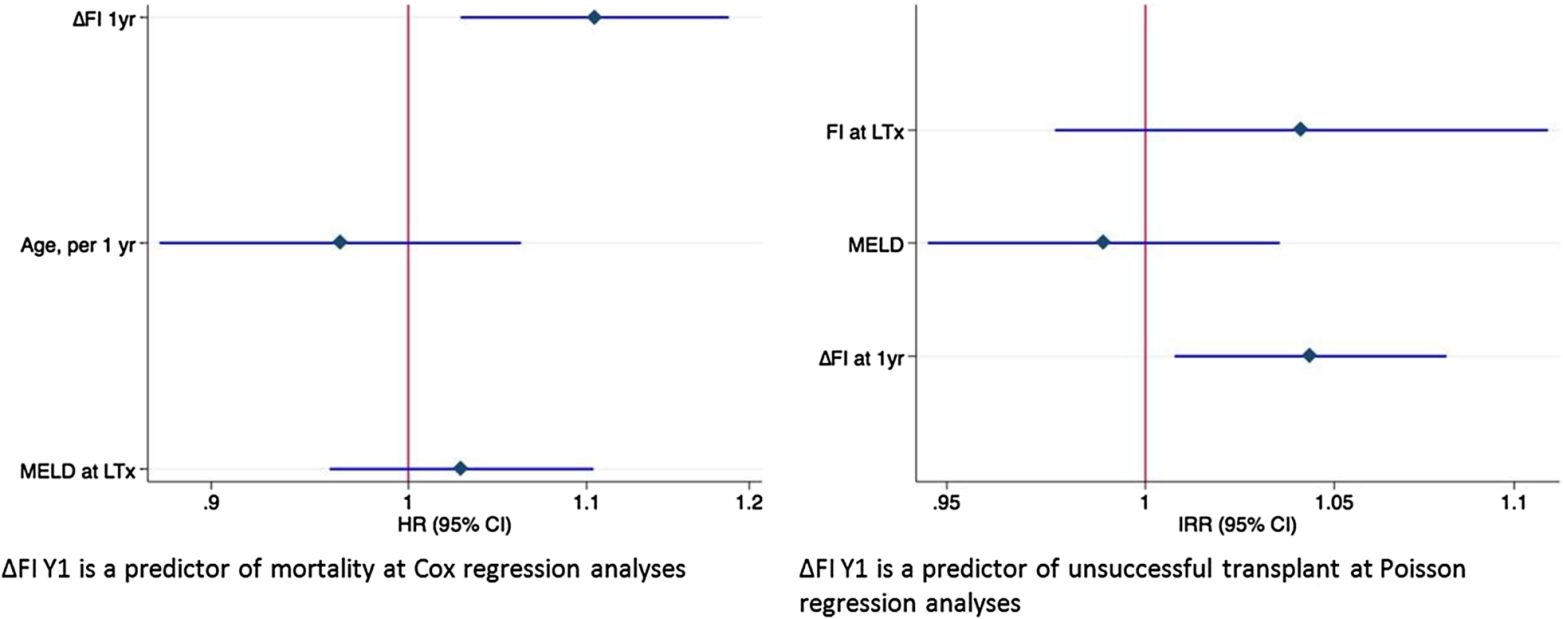
Independent predictors of survival and successful transplant

Figure 2 shows that Δ*FI* at multivariate Cox regression was a predictor of mortality after correction for age and MELD (HR = 1.10, p = 0.006) as well as a predictor of non-optimal transplant at multivariate Poisson regression after correction for age (HR = 1.04, p = 0.01).

#### Frailty index to describe health transition across liver transplant

**Fig. 3:**
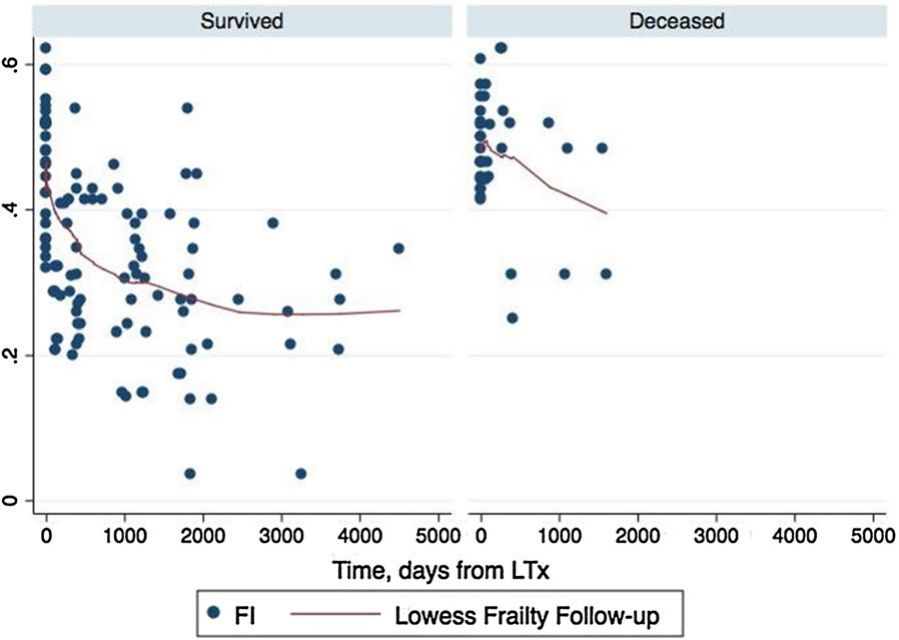
LOWESS smoother: FI time course in survived and deceased. *Red vertical line* represents transplant date

Survivors and deceased patients showed a different pattern FI reduction post-LT. This is visually depicted in Fig. 3 by a LOWESS smoother of the FI in function of time and describes a different health transition across LT in the two groups.

Among the 29 most frail patients at the baseline, during the first year post-LT 10 died, three remained stable and 16 improved (reverted from “most frail” to “frail”). In contrast, among the 18 patients who were “frail” at baseline, 6 did not have enough data to calculate the Y1FI, 10 remained stable and two reverted their condition to “non frail”. Notably, none of them worsened their “frail” status to “most frail”.

Baseline frailty index scores strongly influenced transition probabilities at year 1 (r = 0.37, p = 0.027).

## Discussion

This study validates FI in HIV transplant setting and describes health status transition across the overall LT process.

In the pre-transplant assessment FI measurement (baseline FI) exhibited expected characteristics of the general population with regards to normal distribution, association with age and upper limit ceiling effect [29, 40].

Of interest, baseline FI was conversely associated with DRI. This index can be considered a proxy of the “quality of the graft”. Thus, in our cohort better livers were allocated to patients with a higher FI, suggesting that baseline FI reflected the clinical judgment of vulnerability that surgeons empirically consider when allocating organs, within the respect of urgency criteria established by MELD score.

With regards to criterion validity, baseline “most frail” condition was able to predict mortality and in the post-LT, with 10 out 29 patients died at year 1 between the “most frail” group vs 0 out of 18 among the “frail” one. Moreover, FI at year 1 was able to independently predict mortality.

It must be noticed that in this cohort pre-LT MELD was not associated with neither post-LT mortality nor baseline FI. The MELD provide a parsimonious measure of liver-related health associated with pre-transplant outcome, whereas FI is a copious measure of overall health throughout the transplant process. Thus, we believe that not only the use of FI in the LT setting has the potential to be clinically useful for candidates selection and targeted rehabilitation therapies, but also that it could be of particular interest in the HIV population, where a higher vulnerability is present, comorbidities are more prevalent and where MELD is often less meaningful.

Unfortunately, even though the IRR of the association between optimal transplant and “most frail” condition at the baseline is consistent with the survival analysis, baseline FI was not significantly able to predict optimal transplant. However, its significant association with Y1FI suggests that this could be explained with a lack of statistical power due to the small sample size of this unique cohort.

In addition, we used our FI the health transition across the LT period. As shown in Fig. 3, frailty reduction was present in both deceased and survived patients, but the slope was more evident in the latter group. This picture is consistent with the FI change observed in HIV-negative elderly population where frailty reduction was associated with improved survival [41, 42].

Δ*FI* is an interesting clinical conceptualization given that it allows considering a health status measure as the net change of biological events happening across LT, therefore taking in consideration in the same measure patient vulnerability both in the pre and in the post LT period. Applicability of FI in this clinical setting may also offer a measure of intensity of care giving insight in the large variability of health status in the post-LT.

Δ*FI* beyond year 1 was associated with both mortality and non-optimal transplant outcome, after correction for baseline confounders. Δ *FI* describes health transition across transplant, visually depicted by the LOWESS smoother curve. Mitnitski et al. [16, 43, 44] state that frailty can be considered a proxy of biological age, thus, this findings quantifies the empirical perception of both patients and physicians that LT “puts the biological clock back” to the time before ESLD.

On a clinical standpoint, monitoring FI and delta FI after transplant can be useful. In particular, for patients that experienced less FI improvement more frequent follow-up appointments can be considered or hospital discharge should be delayed.

Some limitations can be acknowledged. The retrospective observational nature of the study avoided the possibility to expand FI variable list, which were close to the minimum number suggested for a FI validation. In particular, our FI lacks of patient-related outcome and geriatric syndrome evaluation usually part of geriatric FI variable lists.

The relatively small sample size of our cohort could be responsible of low statistical power of multivariable analysis not reaching statistical significant value.

Some strength points may also be recognised:

The validation of FI in the transplant setting provide a new biological variable able to capture not only recipient vulnerability and surgical risk but also describe health transition across LT.

Frailty could play a role in a “blended principle model” [1] of organ allocation, as it is able to assess patients’ overall health status and at the same time potential to be a predictor of both mortality and quality of life [23].

This approach also allows to link health status with biological age conceptualization: at an individual basis, LT is a turning point, with different perspective in patients and physicians. Patients often describe LT as a “re-birth”, meaning the biological and psychological aspect related to a new donated life. Along with the obvious psychological benefit of passing from facing death to a longer life expectancy, the health and liver-related symptoms gain after LT reflects in a well-documented improvement in health [45–48]. Physicians often describe LT as a “reverse time” story, somehow putting back the biological clock to the time before ESLD. Both “re-birth”, and “reverse time” refer to the biological age categories, which can be depicted in the frailty assessment. This approach depicting health transition across the transplant period allows interpreting LT as a time reversing event measuring health improvement.

We hope these results may pilot future studies to compare different tools to evaluate frailty in the transplant setting and to expand the validation of FI toward other transplant settings.

## Additional file

**Additional file 1: Figure S1.** Prevalence of Optimal transplant at year 1, year 3 and year 5 visit.

## Abbreviations

AIDS: acquired immunodeficiency syndrome
DeltaFI: delta frailty index
ESLD: end stage liver disease
FI: frailty index
FIY1: frailty index at year 1
FIt0: frailty index at the baseline
HIV: human immunodeficiency virus
HR: hazard ratio
IQR: inter-quartile range
LT: liver transplant
MELD: model for end stage liver disease
MM: multimorbidity
