# Supplementary figures and images for: A frailty index predicts post-liver transplant morbidity and mortality in HIV-positive patients
Source: AIDS Res Ther. 2017 Aug 5;14:37. doi: 10.1186/s12981-017-0163-x (PMC5545092; doi:10.1186/s12981-017-0163-x)

Deaths

MM

Succesfull transplant

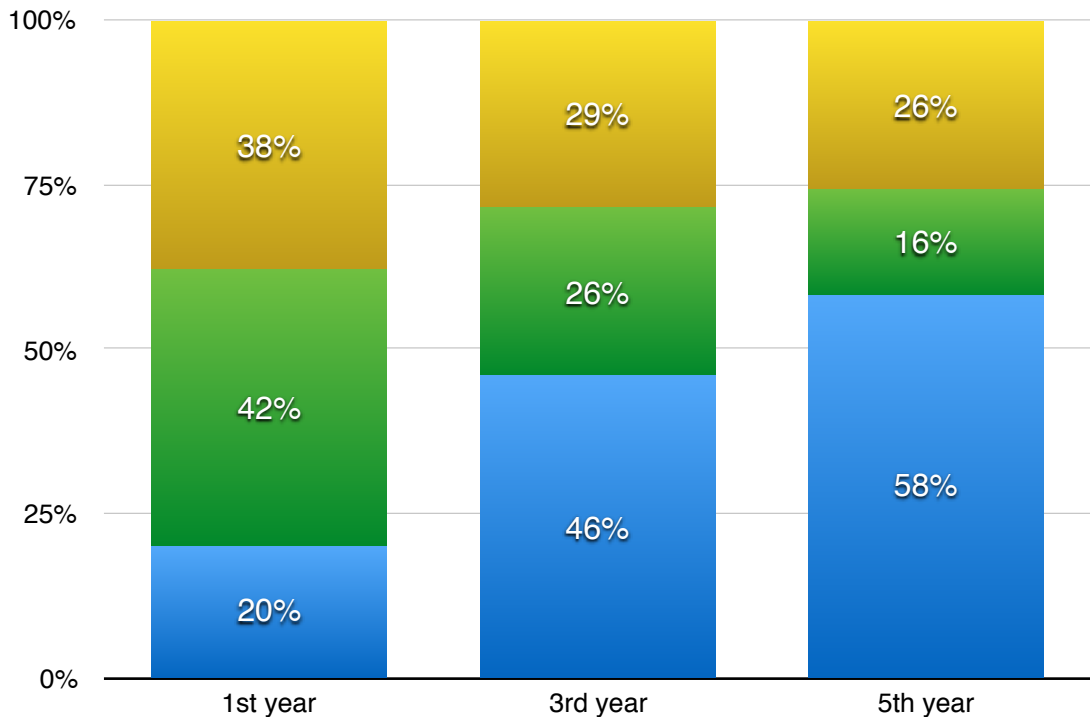

Supplement: Supplementary file 1 — Additional file 1: Figure S1. Prevalence of Optimal transplant at year 1, year 3 and year 5 visit. [file 12981_2017_163_MOESM1_ESM.pdf]
